# Adelmidrol: A New Promising Antioxidant and Anti-Inflammatory Therapeutic Tool in Pulmonary Fibrosis

**DOI:** 10.3390/antiox9070601

**Published:** 2020-07-09

**Authors:** Roberta Fusco, Marika Cordaro, Tiziana Genovese, Daniela Impellizzeri, Rosalba Siracusa, Enrico Gugliandolo, Alessio Filippo Peritore, Ramona D’Amico, Rosalia Crupi, Salvatore Cuzzocrea, Rosanna Di Paola

**Affiliations:** 1 Department of Chemical, Biological, Pharmaceutical and Environmental Sciences, University of Messina, 98166 Messina, Italy; rfusco@unime.it (R.F.); tgenovese@unime.it (T.G.); dimpellizzeri@unime.it (D.I.); rsiracusa@unime.it (R.S.); egugliandolo@unime.it (E.G.); aperitore@unime.it (A.F.P.); rdamico@unime.it (R.D.); 2 Department of Biomedical, Dental and Morphological and Functional Imaging University of Messina, Via Consolare Valeria, 98125 Messina, Italy; cordarom@unime.it; 3 Department of Veterinary Sciences, University of Messina, 98168 Messina, Italy; rcrupi@unime.it; 4 Department of Pharmacological and Physiological Science, Saint Louis University School of Medicine, Saint Louis, MO 63104, USA

**Keywords:** oxidative stress, pulmonary fibrosis, biochemistry

## Abstract

Background: Chronic pulmonary diseases are characterized by airway remodeling due to complex multicellular responses and the production of free oxygen radicals. They lead to a progressive decline of pulmonary functions. Adelmidrol is an analogue of palmitoylethanolamide (PEA), which is a well-known anti-inflammatory and anti-oxidant compound. In this study, we investigated the efficacy of adelmidrol (10 mg/Kg) for bleomycin-induced pulmonary fibrosis in mice. Methods: Bleomycin intratracheal administration was performed on the first day and for the following twenty-one days, mice were treated with adelmidrol (10 mg/Kg). Results: The survival rate and body weight gain were recorded daily. At the end of the experiment, adelmidrol-administered animals showed reduced airway infiltration by inflammatory cells, Myeloperoxidase (MPO) activity, and pro-inflammatory cytokine overexpression (IL,6 IL-1β, TNF-α, and TGF-1β). Moreover, adelmidrol treatment was able to manage the significant incapacity of antioxidants and elevation of the oxidant burden, as shown by the MDA, SOD, and GSH levels and decreased nitric oxide production. It was also able to significantly modulate the JAK2/STAT3 and IκBα/NF-kB pathway. Histologic examination of the lung tissues showed reduced sample injury, mast cell degranulation, chymase activity, and collagen deposition. Conclusions: In sum, our results propose adelmidrol as a therapeutic approach in the treatment of pulmonary fibrosis.

## 1. Introduction

Pulmonary fibrosis is a devastating disease with different causes and has few treatment options [1], resulting in a medium survival of less than four years after diagnosis [2]. The prevailing hypotheses on the pathogenesis of this disease are focused on interactions between the mesenchyma and epithelium, promoting continued fibroblast activation and epithelial injury [3]. The disease is likely multi-factorial in nature, with the dysregulation of multiple pathways, including oxidative stress, chemotaxis, inflammation, tissue remodeling, and wound healing. The lungs of patients exhibit proteinaceous exudate, edema, multinucleated giant cells, and focal reactive hyperplasia of pneumocytes with inflammatory cellular infiltration. Moreover, fibroblastic plugs are found in airspaces. From a molecular point of view, a cytokine and oxidative storm occurs. In particular, by analyzing the immune characteristics of patients, researchers have found irregular pathogenic T cells and activated monocytes producing an aberrant number of cytokines and reactive oxygen species (ROS). The altered alveolar environment involves oxidative stress that is driven by an imbalance between oxidant production and antioxidant defenses.

The bleomycin animal model is usually employed to evaluate the in vivo efficacy of anti-fibrotic and anti-oxidant agents due to the similarities in histological features, such as the mural incorporation of collagen, intra-alveolar buds, and the obliteration of alveolar spaces, found in bleomycin-treated animals and patients with idiopathic pulmonary fibrosis [4].

The intratracheal instillation of bleomycin results in an initial inflammatory reaction marked by the over-expression of pro-inflammatory cytokines and ROS, including hydroxyl radicals and superoxide, interleukin-6, interleukin-1β, tumor necrosis factor-α, superoxide dismutase (SOD), and catalase (CAT), followed by increased levels of pro-fibrotic markers, such as transforming growth factor-β1, fibronectin, and procollagen-1 [5]. The “switch” between the inflammatory and fibrotic stages occurs around day nine after bleomycin instillation [5]. Therefore, drugs administered during the first seven days may predominantly act as anti-inflammatory agents and are considered “preventive or prophylactic”, while drugs administered after seven to ten days could be true anti-fibrotic agents and are considered “therapeutic” [5].

A great number of compounds in different classes, such as antioxidants, angiotensin receptor blockers, anticoagulants, macrolide antibiotics, cytokine antibodies, immunosuppressants, and corticosteroids, have displayed anti-fibrotic effects in the bleomycin model [6]. However, to date, none of the drugs that have made it to clinical trials have been as successful as in bleomycin animal studies.

Several studies have demonstrated that N-acylethanolamines (NAEs), extensively expressed in mammals, are responsible for different physiological functions, including neurotransmission, inflammation, reproduction, appetite, analgesia, and cryoprotection [7]. As well as other NAEs, N-palmitoylethanolamine (PEA) has been widely investigated, showing important neuroprotective, anti-inflammatory, anti-oxidant, and anti-fibrotic effects [8,9,10,11]. PEA is able to stabilize the mitochondrial function, and inhibit mast cell degranulation and anandamide degradation. Its mechanism of action involves IL6 management [10,12,13]. One of the studied ethanolamide derivatives is adelmidrol (N,N’-bis(2-hydroxyethyl)nonanediamide), due to its important anti-inflammatory and anti-oxidant activities. Moreover, it acts by increasing endogenous levels of PEA [14]. It has been found in trace amounts in some whole grains and the human body [15]. Among adelmidrol chemical characteristics, the most important are its hydrophobic and hydrophilic properties that support its solubility in organic and aqueous media [16]. Here, we describe the results of an evaluation of the pharmacological effect of adelmidrol in a bleomycin-induced pulmonary fibrosis model in mice.

## 2. Materials and Methods

### 2.1. Animals

Male CD1 mice (25–30 g, Envigo, Milan, Italy) were accommodated in a controlled location. They received food and water ad libitum. The University of Messina Review Board for animal care (OPBA) approved the study (9 Feb 2017, 137/2017-pr). All in vivo experiments followed the new directives of the USA, Europe, Italy, and the ARRIVE guidelines.

### 2.2. Induction of Lung Injury

Bleomycin administration was performed as previously described [8]. Bleomycin sulphate (1 mg/kg body weight) was delivered by a single intratracheal administration. A volume of 100 μL was injected at end-expiration to guarantee delivery to the distal airways. This was immediately followed by 300 μL of air. One hour after surgery, adelmidrol (10 mg/Kg) was given orally by gavage. The treatment was repeated daily, for 21 days. At the end of the experiment, animals were euthanized and tissues were harvested for further analysis.

### 2.3. Experimental Groups

Mice were randomized into the following experimental groups (*n* = 10):

- Bleomycin + vehicle group. Mice received bleomycin administration and were treated daily with the vehicle (saline);
- Bleomycin + adelmidrol group. Mice received bleomycin administration and were treated daily with adelmidrol (10 mg/Kg);
- Sham + vehicle group. Identical to the bleomycin + vehicle group, but animals received intratracheal instillation of saline (0.9% *w/v*) instead of bleomycin, and were treated daily with the vehicle (saline);
- Sham + adelmidrol group. Identical to the bleomycin + adelmidrol group, but animals received intratracheal instillation of saline (0.9% *w/v*) instead of bleomycin, and were treated daily with adelmidrol (10 mg/Kg).

Mice were euthanized 21 days after bleomycin instillation and tissues were harvested for analyses of injury and inflammation. The dose of adelmidrol was selected based on previous experiments [17,18].

### 2.4. Survival Rate and Body Weight Gain

Mortality and body weight were assessed daily up to 21 days.

### 2.5. Bronchoalveolar Lavage (BAL)

Twenty-one days after bleomycin instillation, mice were euthanized, and the tracheas were cannulated to perform the lavage, as previously described [19]. In total, 0.5 mL Dulbecco’s phosphate-buffered saline (PBS) (GIBCO, Paisley, U.K.) was used. The BAL fluid recovered was spun, the pelleted cells were collected, and the supernatants were stored at −20 °C. In the presence of trypan blue, total BAL cells were counted using a hemocytometer [20]. The total leukocyte number was determined in duplicate using a hemocytometer (in a Burker chamber). For a differential WBC count, a smear was prepared from the cell pellet in bronchoalveolar fluid lavage (BALF) and stained with Wright-Giemsa. After staining, the differential count was carried out by the standard morphological protocol under a light microscope [21].

### 2.6. Measurement of Lung Edema

After 21 days of injections of bleomycin, wet lung weights were recorded. The lungs were subsequently dried for 48 h at 180 °C and then weighed again. The water content of the lungs was calculated as the ratio of wet:dry weight of the tissue.

### 2.7. Histological Examination

Lung tissue samples were collected 21 days from bleomycin injection. After fixing the tissues in buffered formaldehyde solution (10% in PBS), histological sections were stained with haematoxylin and eosin and evaluated using an Axiovision Zeiss (Milan, Italy) microscope. The severity of lung fibrosis was scored on a scale from 0 to 8, as already published [8]. Lung sections were stained with toluidine blue to enumerate mast cells [22] and with Masson’s trichrome for fibrosis [23].

### 2.8. Measurement of Chymase Activity

Lung tissues were homogenized and the chymase activity was measured in the supernatant according to the method described by Pasztor et al. (1991) [24,25].

### 2.9. MPO Assay

MPO activity was determined as already described [8,26]. It was defined as the quantity of enzyme degrading 1 μmol of peroxide per min at 37 °C and was expressed in units per gram wet tissue weight.

### 2.10. Soluble Collagen Assay

The Sircol Soluble Collagen Assay (Biocolor, Newtownabbey, Northern Ireland) was performed following the manufacturer’s instructions [27].

### 2.11. Enzyme-Linked Immunosorbent Assays (ELISA)

The bronchoalveolar fluid lavage (BALF) IL,6 IL-1β, TNF-α, and TGF-1β levels were detected by using ELISA kits (Dakewe, Shenzhen, China; Biosource International, Camarillo, CA) [28,29,30]. Levels of IL6 IL-1β, TNF-α, and TGF-1β in lung tissues were measured by using ELISA kits (Calbiochem-Novabiochem Corporation, USA) [31,32].

### 2.12. Nitric Oxide (NO) Analysis

The pulmonary production of NO in the BALF was analyzed with a nitrate/nitrite colorimetric assay [33]. The absorbance was measured at 550 nm using a plate absorbance reader (Bio-Tek Instruments, Inc.).

### 2.13. Measurement of Oxidative Stress

The malondialdehyde (MDA) [34], glutathione (GSH), and SOD levels in the lung tissues were measured using activity assay kits (Nanjing Jiancheng Bioengineering Institute) [28].

### 2.14. Western Blot Analysis

Western blots were performed as described in our previous studies [35]. Briefly, lung tissues from each mouse were suspended in an extraction’s buffer containing 0.15 µM pepstatin A, 0.2 mM phenylmethylsulfonyl fluoride (PMSF), 1 mM sodium orthovanadate, and 20 µM leupeptin; homogenized at the highest setting for 2 min; and centrifuged at 1000× *g* for 10 min at 4 °C. Supernatants contain the cytosolic fractions, while the pellets represent the nuclear ones. Pellets were re-suspended in a second buffer containing 150 mM sodium chloride (NaCl), 1% Triton X-100, 1 mM ethylene glycol tetraacetic acid (EGTA), 10 mM tris-chloridric acid (HCl) pH 7.4, 0.2 mM PMSF, 1 mM Ethylenediaminetetraacetic acid (EDTA), 0.2 mM sodium orthovanadate, and 20 µm leupeptin. After centrifugation at 4 °C and 15.000 g for 30 min, the nuclear proteins containing the supernatants were stored at −80 °C for further analysis. Specific primary antibody:anti-IkBα (1:1000, Santa Cruz Biotechnology) or anti-NF-kB p65 (1:1000; Santa Cruz Biotechnology) were mixed in 1× PBS, 5% *w/v* non-fat dried milk, and 0.1% Tween-20, and incubated at 4 °C, overnight. Following this, blots were incubated with peroxidase-conjugated bovine anti-mouse IgG secondary antibody or peroxidase-conjugated goat anti-rabbit IgG (1:2000, Jackson Immuno Research) for 1 h at room temperature. To verify that membranes were loaded with equal amounts of protein, they were also incubated with the antibody against laminin (1:1000; Santa Cruz Biotechnology) and GADPH (1: 1000; Santa Cruz Biotechnology). Signals were detected with enhanced chemiluminescence detection system reagent, according to the manufacturer’s instructions (Super- Signal West Pico Chemiluminescent Substrate, Pierce). The relative expression of the protein bands was quantified by densitometry with Bio-Rad ChemiDoc XRS software and standardized tob-actin levels. Images of blot signals (8-bit/600-dpi) were imported to analysis software (Image Quant TL, v2003).

### 2.15. Materials

All compounds used in this study were purchased from Sigma-Aldrich Company Ltd. (Milan, Italy) and were of the highest commercial grade available. Adelmidrol was obtained from Epitech Group SpA. Adelmidrol is classified by the WHO as an INN (International Nonproprietary Name = safe substance).

### 2.16. Statistical Evaluation

All values in the figures and text are expressed as the mean ± standard error of the mean (SEM) of N number of animals. The results were analyzed by one-way ANOVA followed by a Bonferroni post-hoc test for multiple comparisons. A *p*-value < 0.05 was considered significant. * *p* < 0.05 vs. sham+vehicle, ^#^ *p* < 0.05 vs. vehicle, ** *p* < 0.01 vs. sham+vehicle, ^##^ *p* < 0.01 vs. vehicle, *** *p* < 0.001 vs. sham+vehicle, and ^###^ *p* < 0.001 vs. vehicle.

## 3. Results

### 3.1. Adelmidrol Exerts Anti-Inflammatory Effects in a Pulmonary Fibrosis Model

**Figure 1:**
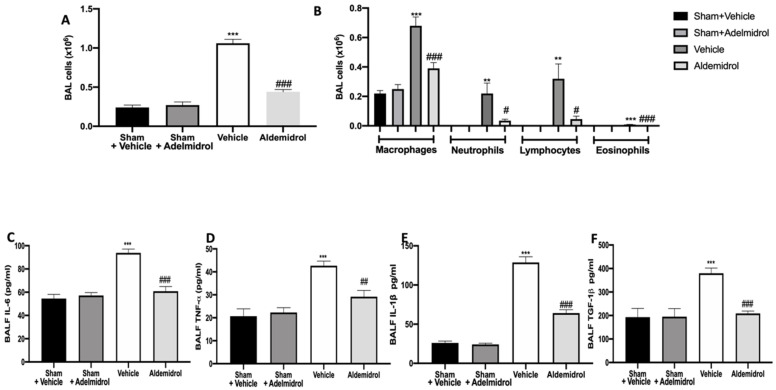
Effect of adelmidrol on cell infiltration, MPO activity, and proinflammatory cytokine expression in bronchoalveolar lavage fluid: Total cells (**A**); macrophages, neutrophils, lymphocytes, and eosinophils (**B**); IL6 (**C**); TNF-α (**D**); IL-1β (**E**); and TGF-1β (**F**) expressions in bronchoalveolar fluid lavage (BALF). ^#^ *p* < 0.05 vs. vehicle, ** *p* < 0.01 vs. sham + vehicle, ^##^ *p* < 0.01 vs. vehicle, *** *p* < 0.001 vs. sham + vehicle, and ^###^ *p* < 0.001 vs. vehicle.

Twenty-one days after bleomycin instillation, adelmidrol administration reduced the number of inflammatory cells in the bronchoalveolar lavage fluid compared to the vehicle-treated mice (Figure 1A). In particular, we evaluated macrophages, neutrophils, lymphocytes, and eosinophils, observing a significant rise in cell numbers in bronchoalveolar lavage collected from vehicle-treated animals compared to the sham groups. Adelmidrol treatment was able to reduce the airway infiltration by inflammatory cells (Figure 1B). An analysis of cytokine expressions in the bronchoalveolar lavage fluid showed that pulmonary fibrosis increased IL,6 IL-1β, TNF-α, and TGF-1β expressions compared to the sham groups. In the bronchoalveolar lavage fluid collected from adelmidrol-treated mice, a reduced expression of IL,6 IL-1β, TNF-α, and TGF-1β was detected (Figure 1C–F).

### 3.2. Adelmidrol Modulates the Lung Production of IL6, TNF-α, and IL-1β and Oxidative Stress Induced by Pulmonary Fibrosis

**Figure 2:**
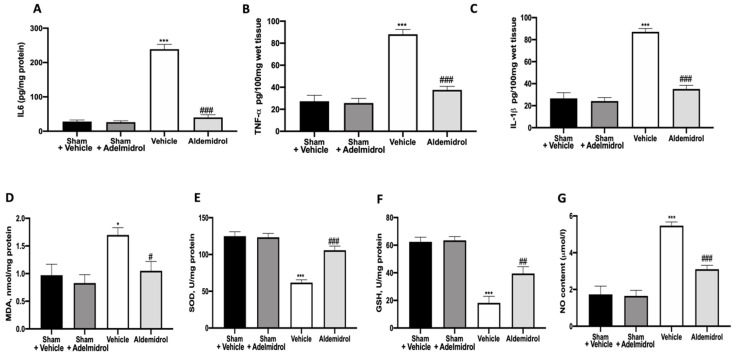
Effect of adelmidrol on lung tissue cytokine expression and oxidative stress: IL6 (**A**), TNF-α (**B**), and IL-1β (**C**) expressions in lungs; malondialdehyde (MDA) (**D**), superoxide dismutase (SOD) (**E**), and glutathione (GSH) (**F**) levels; and nitric oxide (NO) content (**G**). * *p* < 0.05 vs. sham + vehicle, ^#^ *p* < 0.05 vs. vehicle, ^##^ *p* < 0.01 vs. vehicle, *** *p* < 0.001 vs. sham + vehicle, and ^###^ *p* < 0.001 vs. vehicle.

To test whether adelmidrol may modulate the inflammatory process through regulation of the secretion of cytokines, we analyzed the lung tissue levels of the pro-inflammatory cytokines IL6, TNF-α, and IL-1β. A substantial increase in IL6, TNF-α, and IL-1β formation was observed in lung samples taken from vehicle-treated mice when compared with sham-operated animals. In contrast, there was a significant inhibition of IL6, TNF-α, and IL-1β in instilled-mice treated with adelmidrol (Figure 2A–C). MDA, SOD, and GSH levels were evaluated twenty-one days after bleomycin induction. Vehicle-treated mice showed increased MDA levels, while SOD and GSH were decreased, compared to the sham groups. Once again, adelmidrol administration was able to reduce the oxidative stress parameters analyzed (Figure 2D–F). NO, an endogenous short-lived free radical, has a crucial role in the development of pulmonary fibrosis. Vehicle-treated mice exhibited increased NO production in the bronchioalveolar lavage fluid compared to the sham groups. Adelmidrol administration decreased the NO content (Figure 2G).

### 3.3. Adelmidrol Manages the STAT3 and NF-kB Pathway

**Figure 3:**
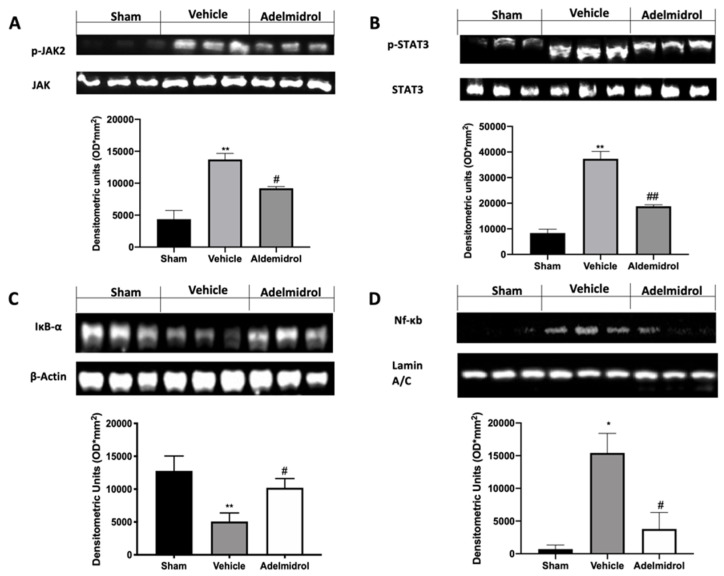
Effect of adelmidrol on p-JAK2, p-STAT3, Ikb-α, and NF-kB expression: Western blot analysis of p-JAK2 (**A**), p-STAT3 (**B**), Ikb-α (**C**), and NF-kB (**D**). * *p* < 0.05 vs. sham + vehicle, ^#^ *p* < 0.05 vs. vehicle, ** *p* < 0.01 vs. sham + vehicle, ^##^ *p* < 0.01 vs. vehicle.

We investigated one of the key inflammatory pathways induced by pulmonary fibrosis: JAK2/STAT3 and Ikb-α/NF-kB systems. Phospho-janus kinase (p-JAK2) expression levels, monitored by Western blotting, were considerably increased in tissue collected from vehicle-treated mice compared to the sham animals. Lung tissues harvested from adelmidrol-treated mice showed a reduced p-JAK2 expression (Figure 3A). Moreover, traumatic brain injury induced increased STAT3 phosphorylation in vehicle-treated mice, while it was remarkably reduced in samples from adelmidrol-administered animals (Figure 3B). Ikb-α basal expression was observed in lungs collected from sham mice, while bleomycin instillation reduced it (Figure 3C). In addition, NF-kB expression in the nucleus was enhanced in vehicle-treated mice compared to the sham. Treatment with adelmidrol reduced both Ikb-α cytosol expression and NF-kB expression in the nucleus (Figure 3D).

### 3.4. Adelmidrol Reduced the Mortality Rate and Improved the Histopathological Score

**Figure 4:**
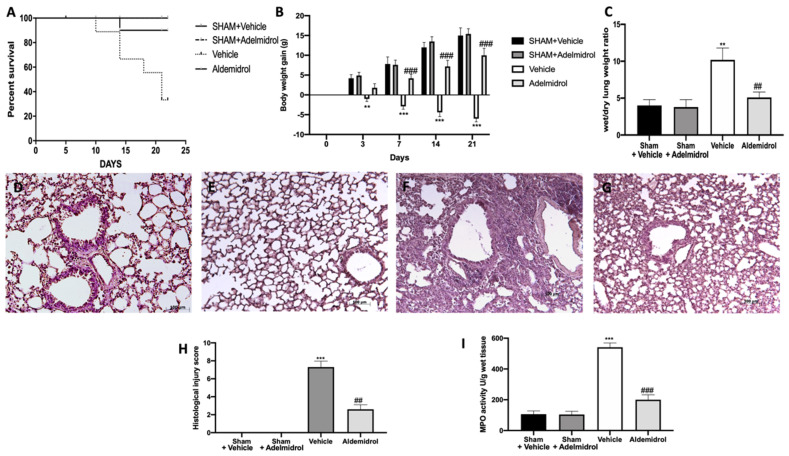
Effect of adelmidrol on the survival rate, body weight, lung edema, histological changes, MPO activity, and fibrosis: Survival rate (**A**); body weight gain (**B**); wet/dry lung weight ratio (**C**); hematoxylin and eosin staining of sham+vehicle (**D**), sham+adelmidrol (**E**), vehicle (**F**), and adelmidrol (**G**); histological injury score (**H**); MPO activity (**I**). ** *p* < 0.01 vs. sham+vehicle, ^##^ *p* < 0.01 vs. vehicle, *** *p* < 0.001 vs. sham+vehicle, and ^###^ *p* < 0.001 vs. vehicle.

Pulmonary fibrosis is associated with an important morality rate and body weight decrease. Adelmidrol administration was able to increase the survival rate (Figure 4A) and the body weight gain (Figure 4B) compared to the vehicle-treated mice. Moreover, it decreased the lung edema, as shown by the ratio of wet/dry weight of the tissue (Figure 4C). Histologic examination of the lungs revealed the abundant tissue damage and extracellular matrix deposition in tissues collected from vehicle-treated mice (Figure 4F,G), compared to the sham groups (Figure 4D,E,H). Adelmidrol treatment significantly reduced the lung injury (Figure 4G,H). In order to evaluate the activity of neutrophils, we performed the MPO assay. Pulmonary fibrosis increased the MPO activity, while adelmidrol administration reduced it (Figure 4I).

### 3.5. Adelmidrol Reduced Mast Cell Degranulation and Lung Fibrotic Changes

**Figure 5:**
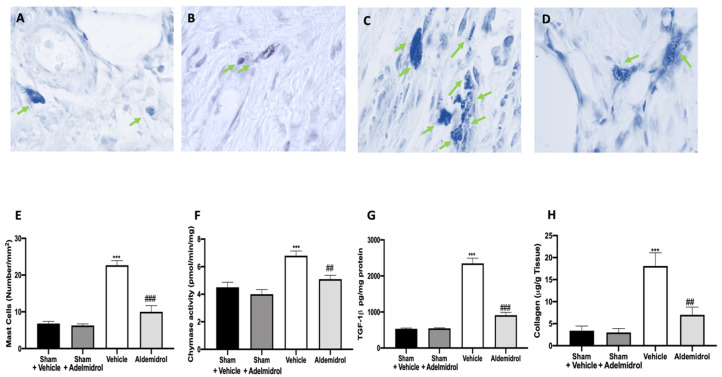
Effect of adelmidrol on mast cell analysis: Sham+vehicle (**A**), sham+adelmidrol (**B**), vehicle (**C**), adelmidrol (**D**); mast cell count (**E**); chymase activity (**F**); TGF-1β (**G**) expression; collagen deposition (**H**) in lungs. ^##^ *p* < 0.01 vs. vehicle, *** *p* < 0.001 vs. sham + vehicle, and ^###^ *p* < 0.001 vs. vehicle.

**Figure 6:**
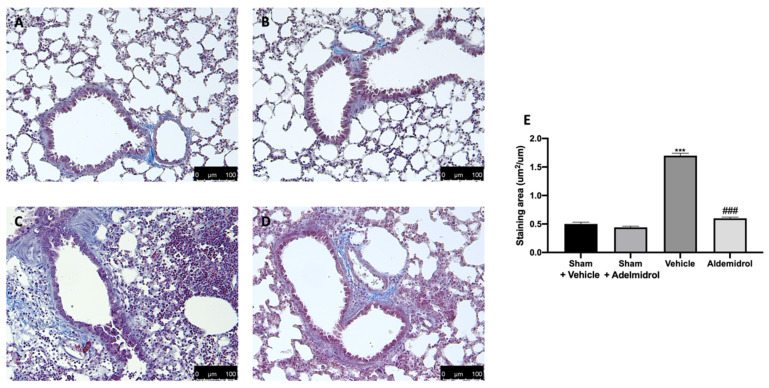
Effect of adelmidrol on Masson’s trichrome staining: Sham+vehicle (**A**), sham+adelmidrol (**B**), vehicle (**C**), adelmidrol (**D**), and staining area (**E**). *** *p* < 0.001 vs. sham+vehicle, and ^###^ *p* < 0.001 vs. vehicle.

Toluidine blue staining was performed on lung tissues from bleomycin-injected animals in order to evaluate mast cell activation and recruitment in the inflamed tissues. No mast cells were detected in tissues from sham-treated animals (Figure 5A,B,E), while vehicle-treated animals displayed an increased number of infiltrating mast cells (Figure 5C,E). Adelmidrol (Figure 5D,E) treatment reduced the recruitment in lungs. Bleomycin administration also increased chymase activity in lungs, while adelmidrol was able to reduce it (Figure 5F). Severe pulmonary injury produced by bleomycin in lungs from vehicle-treated mice resulted in extensive TGF-1β expression and collagen deposition compared to the sham groups (Figure 5G,H). In contrast, adelmidrol treatment reduced this deposition and TGF-1β expression in the lungs (Figure 5H,G). We also evaluated fibrosis by Masson’s trichrome staining. Tissues harvested from vehicle-treated mice showed increased collagen deposition around airway walls and blood vessels (Figure 6C,H) compared to the sham groups (Figure 6A,B,E). Adelmidrol treatment reduced the bleomycin-induced collagen deposition after analysis in the image-processing system (Figure 6D,E).

## 4. Discussion

Our experiments examined the effects of adelmidrol administration on bleomycin-induced pulmonary fibrosis: in particular, this study indicates that adelmidrol has important anti-inflammatory and anti-oxidant properties, leading to a reduced expression of pro-inflammatory cytokines and the production of free oxygen radicals, a restored expression of anti-oxidant enzymes in the bronchoalveolar lavage fluid and lung tissues, and reduced collagen deposition. Adelmidrol, a member of the aliamide family, has similar anti-nociceptive and anti-inflammatory proprieties of PEA [19,36,37,38,39]. In fact, as a PEA synthetic analogue, adelmidrol increases endogenous levels of PEA [14]. It is well-described that the intratracheal instillation of bleomycin causes lung damage, primarily to alveolar epithelial cells, and then results in lymphocytic and neutrophilic infiltration and pan-alveolitis [40]. Despite bleomycin animal models of pulmonary fibrosis not completely recapitulating the SARS-CoV2 pathology, because in this model, the fibrotic response follows acute lung injury rather than the de-novo progressive fibrosis, bleomycin models may still be useful in studying fibrosis as a complication of COVID-19. Twenty-one days after bleomycin administration, bronchoalveolar lavage fluid collected from vehicle-treated mice showed significant total leukocyte airway infiltration, as well as increased lymphocyte, macrophage, neutrophil, and eosinophil numbers and enhanced MPO activity. It is well-known that many tissue responses to inflammatory injuries are activated and managed by cytokines and oxidative stress [41,42]. Adelmidrol administration was able to reduce inflammatory cell infiltration in the bronchoalveolar lavage and chemokines and cytokines increased the expression induced by pulmonary injury. ELISA analysis of lung tissues also showed a reduced expression of the same cytokines (IL6, TNF-α, and IL-1β) already investigated in the bronchoalveolar lavage fluid, confirming the adelmidrol mechanism of action. Bleomycin toxicity is also related to free oxygen radical production and the enhancement of oxidative stress [43]. In lungs, several antioxidant enzyme systems, including CAT, SOD, and glutathione peroxidase (GPxs), have been described [44]. The overexpression of these enzymes is protective against fibrosis [45,46]. Moreover, bleomycin instillation binds molecular oxygen and Fe (II), producing ROS mediators and causing lipid peroxidation [47]. Adelmidrol treatment reduced the significant disability of antioxidants and elevation of the oxidant burden, as shown by the SOD activity and MDA and GSH levels, induced by bleomycin instillation [28,31,48]. Mice subjected to an experimental model of pulmonary fibrosis showed NO over-production [49,50,51], similar to patients affected by this pathology [52]. However, no modifications in other NOS levels have been detected [52]. In the current study, we showed that adelmidrol administration was able to reduce NO production. Our results provide evidence that the anti-inflammatory and antifibrotic effects of adelmidrol are mediated by reduced cytokine expressions, resulting in the downregulation of TGF-1β, oxidative stress, and NO production. This increased expression of chemokines, cytokines, and inflammatory mediators also induces changes in transcription factors. JAKs are receptor-associated tyrosine kinases with central roles in cytokine and growth factor signaling. Like other receptor-associated tyrosine kinases, cytokine binding induces the autophosphorylation and activation of JAK kinases [53]. In turn, JAK kinases recruit and phosphorylate signal transducer and activator of transcription (STAT) proteins. Upon activation, STATs dimerize and translocate to the nucleus, where they activate the transcription of several target genes [53]. Alterations in JAK2 signaling cause profound changes in the cellular response to cytokine stimulation. TGF-1β signaling induces the phosphorylation and activation of JAK2, which then interacts and phosphorylates STAT3 to induce fibrotic responses [54]. Bleomycin intratracheal administration enhanced p-JAK2 and p-STAT3 levels, while adelmidrol treatment was able to reduce the phosphorylation of both proteins. Dual JAK2/STAT3 inhibition was more effective for inhibiting both of these cellular transitions and lung fibrosis than the individual inhibition of JAK2 or STAT3, which implies synergistic and independent roles of these proteins in pulmonary fibrosis. Moreover, it has been described that IL6, TNF-α, and IL-1β lead to activation of the NF-kB pathway, which is the major controller of pulmonary fibrosis and epithelial–mesenchymal transition, in response to chronic inflammation [55]. It is normally stored in cytoplasm bound to the IκBα inhibitor. Once activated by particular stimuli, such as inflammation, infection, and oxidative stress, it is phosphorylated by IκB kinase, resulting in NF-kB release and nuclear translocation of the subunit p65 [56]. Adelmidrol treatment significantly reduced NF-kB nuclear expression. Pulmonary fibrosis also causes severe modifications of the lung architecture [57], leading to compromised gas exchange and respiratory failure. Adelmidrol-treated animals exhibited an increase survival rate, body weight gain, and reduced lung edema induced by bleomycin administration. Mast cells are also known to mediate fibrogenic events [58]. Moreover, abundant numbers of mast cells have been verified in the lungs of patients with different forms of fibrosis [59,60,61,62,63]. They contain many profibrotic molecules, including chymase, tryptase, leukotrienes, histamine, and renin [64]. Therefore, the degranulation and activation of mast cells may contribute to lung fibrosis [65]. Of particular relevance is chymase secreted by mast cells [66]. It is a chymotrypsin serine protease that may activate the latent TGF-1β [65]. TGF-1β is a fibrogenic cytokine [67] and has been described to have a key role in the development of pulmonary fibrosis [68]; in fact, its expression has been found to be increased in patients and a lung fibrosis mouse model [69,70]. TGF-β stimulates fibroblast differentiation into myofibroblasts [68,71]. Therefore, chymase may induce fibroblast activation and increase collagen synthesis through TGF-1β activation. Mast cell degranulation may indirectly or/and directly contribute to lung fibrosis [22]. Lungs harvested from adelmidrol-treated animals showed reduced histological lung injury and MPO activity compared to those from vehicle-treated mice. Adelmidrol, by stabilizing mast cell degranulation, reduced chymase activity, TGF-1β expression, and collagen deposition.

## 5. Conclusions

In conclusion, our data propose adelmidrol as a therapeutic approach in the treatment of pulmonary fibrosis. We have demonstrated its ability to downregulate the over-production of pro-inflammatory cytokines, in particular, IL6 and its anti-fibrotic and anti-oxidant properties.

## References

[1] King T.E., Pardo A., Selman M. (2011). Idiopathic pulmonary fibrosis. Lancet.

[2] Datta A., Scotton C.J., Chambers R.C. (2011). Novel therapeutic approaches for pulmonary fibrosis. Br. J. Pharmacol..

[3] Usuki J., Fukuda Y. (1995). Evolution of three patterns of intra-alveolar fibrosis produced by bleomycin in rats. Pathol. Int..

[4] Shi K., Jiang J., Ma T., Xie J., Duan L., Chen R., Song P., Yu Z., Liu C., Zhu Q. (2014). Pathogenesis pathways of idiopathic pulmonary fibrosis in bleomycin-induced lung injury model in mice. Respir. Physiol. Neurobiol..

[5] Chaudhary N.I., Schnapp A., Park J.E. (2006). Pharmacologic differentiation of inflammation and fibrosis in the rat bleomycin model. Am. J. Respir. Crit. Care Med..

[6] Moeller A., Ask K., Warburton D., Gauldie J., Kolb M. (2008). The bleomycin animal model: A useful tool to investigate treatment options for idiopathic pulmonary fibrosis?. Int. J. Biochem. Cell Biol..

[7] Schmid H.H., Berdyshev E.V. (2002). Cannabinoid receptor-inactive N-acylethanolamines and other fatty acid amides: Metabolism and function. Prostaglandins Leukot. Essent. Fatty Acids.

[8] Di Paola R., Impellizzeri D., Fusco R., Cordaro M., Siracusa R., Crupi R., Esposito E., Cuzzocrea S. (2016). Ultramicronized palmitoylethanolamide (PEA-um((R))) in the treatment of idiopathic pulmonary fibrosis. Pharmacol. Res..

[9] Fusco R., Gugliandolo E., Campolo M., Evangelista M., Di Paola R., Cuzzocrea S. (2017). Effect of a new formulation of micronized and ultramicronized N-palmitoylethanolamine in a tibia fracture mouse model of complex regional pain syndrome. PLoS ONE.

[10] Di Paola R., Cordaro M., Crupi R., Siracusa R., Campolo M., Bruschetta G., Fusco R., Pugliatti P., Esposito E., Cuzzocrea S. (2016). Protective Effects of Ultramicronized Palmitoylethanolamide (PEA-um) in Myocardial Ischaemia and Reperfusion Injury in VIVO. Shock.

[11] Impellizzeri D., Peritore A.F., Cordaro M., Gugliandolo E., Siracusa R., Crupi R., D’Amico R., Fusco R., Evangelista M., Cuzzocrea S. (2019). The neuroprotective effects of micronized PEA (PEA-m) formulation on diabetic peripheral neuropathy in mice. FASEB J..

[12] Cordaro M., Impellizzeri D., Bruschetta G., Siracusa R., Crupi R., Di Paola R., Esposito E., Cuzzocrea S. (2016). A novel protective formulation of Palmitoylethanolamide in experimental model of contrast agent induced nephropathy. Toxicol. Lett..

[13] Impellizzeri D., Bruschetta G., Cordaro M., Crupi R., Siracusa R., Esposito E., Cuzzocrea S. (2014). Micronized/ultramicronized palmitoylethanolamide displays superior oral efficacy compared to nonmicronized palmitoylethanolamide in a rat model of inflammatory pain. J. Neuroinflamm..

[14] Petrosino S., Puigdemont A., Della Valle M.F., Fusco M., Verde R., Allara M., Aveta T., Orlando P., Di Marzo V. (2016). Adelmidrol increases the endogenous concentrations of palmitoylethanolamide in canine keratinocytes and down-regulates an inflammatory reaction in an in vitro model of contact allergic dermatitis. Vet. J..

[15] Ostardo E., Impellizzeri D., Cervigni M., Porru D., Sommariva M., Cordaro M., Siracusa R., Fusco R., Gugliandolo E., Crupi R. (2018). Urology Study, G. Adelmidrol + sodium hyaluronate in IC/BPS or conditions associated to chronic urothelial inflammation. A translational study. Pharmacol. Res..

[16] De Filippis D., D’Amico A., Cinelli M.P., Esposito G., Di Marzo V., Iuvone T. (2009). Adelmidrol, a palmitoylethanolamide analogue, reduces chronic inflammation in a carrageenin-granuloma model in rats. J. Cell Mol. Med..

[17] Di Paola R., Fusco R., Impellizzeri D., Cordaro M., Britti D., Morittu V.M., Evangelista M., Cuzzocrea S. (2016). Adelmidrol, in combination with hyaluronic acid, displays increased anti-inflammatory and analgesic effects against monosodium iodoacetate-induced osteoarthritis in rats. Arthritis Res. Ther..

[18] Cordaro M., Impellizzeri D., Gugliandolo E., Siracusa R., Crupi R., Esposito E., Cuzzocrea S. (2016). Adelmidrol, a Palmitoylethanolamide Analogue, as a New Pharmacological Treatment for the Management of Inflammatory Bowel Disease. Mol. Pharmacol..

[19] Genovese T., Esposito E., Mazzon E., Di Paola R., Meli R., Bramanti P., Piomelli D., Calignano A., Cuzzocrea S. (2008). Effects of palmitoylethanolamide on signaling pathways implicated in the development of spinal cord injury. J. Pharmacol. Exp. Ther..

[20] Impellizzeri D., Talero E., Siracusa R., Alcaide A., Cordaro M., Maria Zubelia J., Bruschetta G., Crupi R., Esposito E., Cuzzocrea S. (2015). Protective effect of polyphenols in an inflammatory process associated with experimental pulmonary fibrosis in mice. Br. J. Nutr..

[21] Saadat S., Beheshti F., Askari V.R., Hosseini M., Mohamadian Roshan N., Boskabady M.H. (2019). Aminoguanidine affects systemic and lung inflammation induced by lipopolysaccharide in rats. Respir. Res..

[22] Shimbori C., Upagupta C., Bellaye P.-S., Ayaub E.A., Sato S., Yanagihara T., Zhou Q., Ognjanovic A., Ask K., Gauldie J. (2019). Mechanical stress-induced mast cell degranulation activates TGF-β1 signalling pathway in pulmonary fibrosis. Thorax.

[23] Zhang C., Zhang L.H., Wu Y.F., Lai T.W., Wang H.S., Xiao H., Che L.Q., Ying S.M., Li W., Chen Z.H. (2016). Suhuang antitussive capsule at lower doses attenuates airway hyperresponsiveness, inflammation, and remodeling in a murine model of chronic asthma. Sci. Rep..

[24] Pasztor M., Fischer J., Nagy Z., Sohar I. (1991). Proteolytic enzyme activities in rat peritoneal exudate. Acta Biol. Hung..

[25] Tomimori Y., Muto T., Saito K., Tanaka T., Maruoka H., Sumida M., Fukami H., Fukuda Y. (2003). Involvement of mast cell chymase in bleomycin-induced pulmonary fibrosis in mice. Eur. J. Pharmacol..

[26] Mullane K.M., Kraemer R., Smith B. (1985). Myeloperoxidase activity as a quantitative assessment of neutrophil infiltration into ischemic myocardium. J. Pharmacol. Methods.

[27] Conte E., Fagone E., Gili E., Fruciano M., Iemmolo M., Pistorio M.P., Impellizzeri D., Cordaro M., Cuzzocrea S., Vancheri C. (2016). Preventive and therapeutic effects of thymosin beta4 N-terminal fragment Ac-SDKP in the bleomycin model of pulmonary fibrosis. Oncotarget.

[28] Zhang J., Cui R., Feng Y., Gao W., Bi J., Li Z., Liu C. (2018). Serotonin Exhibits Accelerated Bleomycin-Induced Pulmonary Fibrosis through TPH1 Knockout Mouse Experiments. Mediators Inflamm..

[29] Yasui H., Gabazza E.C., Tamaki S., Kobayashi T., Hataji O., Yuda H., Shimizu S., Suzuki K., Adachi Y., Taguchi O. (2001). Intratracheal administration of activated protein C inhibits bleomycin-induced lung fibrosis in the mouse. Am. J. Resp. Crit. Care.

[30] Atzori L., Chua F., Dunsmore S., Willis D., Barbarisi M., McAnulty R., Laurent G. (2004). Attenuation of bleomycin induced pulmonary fibrosis in mice using the heme oxygenase inhibitor Zn-deuteroporphyrin IX-2, 4-bisethylene glycol. Thorax.

[31] Liu M.H., Lin A.H., Ko H.K., Perng D.W., Lee T.S., Kou Y.R. (2017). Prevention of Bleomycin-Induced Pulmonary Inflammation and Fibrosis in Mice by Paeonol. Front. Physiol..

[32] Luzina I.G., Lockatell V., Todd N.W., Kopach P., Pentikis H.S., Atamas S.P. (2015). Pharmacological In Vivo Inhibition of S-Nitrosoglutathione Reductase Attenuates Bleomycin-Induced Inflammation and Fibrosis. J. Pharmacol. Exp. Ther..

[33] Liu L., Lu W., Ma Z., Li Z. (2012). Oxymatrine attenuates bleomycin-induced pulmonary fibrosis in mice via the inhibition of inducible nitric oxide synthase expression and the TGF-β/Smad signaling pathway. Int. J. Mol. Med..

[34] Ohkawa H., Ohishi N., Yagi K. (1979). Assay for lipid peroxides in animal tissues by thiobarbituric acid reaction. Anal. Biochem..

[35] Gugliandolo E., Fusco R., Biundo F., D’Amico R., Benedetto F., Di Paola R., Cuzzocrea S. (2017). Palmitoylethanolamide and Polydatin combination reduces inflammation and oxidative stress in vascular injury. Pharmacol. Res..

[36] Aloe L., Leon A., Levi-Montalcini R. (1993). A proposed autacoid mechanism controlling mastocyte behaviour. Agents Actions.

[37] Costa B., Comelli F., Bettoni I., Colleoni M., Giagnoni G. (2008). The endogenous fatty acid amide, palmitoylethanolamide, has anti-allodynic and anti-hyperalgesic effects in a murine model of neuropathic pain: Involvement of CB(1), TRPV1 and PPARgamma receptors and neurotrophic factors. Pain.

[38] De Filippis D., Luongo L., Cipriano M., Palazzo E., Cinelli M.P., de Novellis V., Maione S., Iuvone T. (2011). Palmitoylethanolamide reduces granuloma-induced hyperalgesia by modulation of mast cell activation in rats. Mol. Pain.

[39] Esposito E., Paterniti I., Mazzon E., Genovese T., Di Paola R., Galuppo M., Cuzzocrea S. (2011). Effects of palmitoylethanolamide on release of mast cell peptidases and neurotrophic factors after spinal cord injury. Brain Behav. Immun..

[40] Janick-Buckner D., Ranges G.E., Hacker M.P. (1989). Alteration of bronchoalveolar lavage cell populations following bleomycin treatment in mice. Toxicol. Appl. Pharmacol..

[41] Toews G.B. (2001). Cytokines and the lung. Eur. Respir. J. Suppl..

[42] Coward W.R., Saini G., Jenkins G. (2010). The pathogenesis of idiopathic pulmonary fibrosis. Ther. Adv. Respir. Dis..

[43] Hay J., Shahzeidi S., Laurent G. (1991). Mechanisms of bleomycin-induced lung damage. Arch. Toxicol..

[44] Day B.J. (2008). Antioxidants as potential therapeutics for lung fibrosis. Antioxid. Redox Signal..

[45] Gao F., Kinnula V.L., Myllarniemi M., Oury T.D. (2008). Extracellular superoxide dismutase in pulmonary fibrosis. Antioxid. Redox Signal..

[46] Hagen T.M., Brown L.A., Jones D.P. (1986). Protection against paraquat-induced injury by exogenous GSH in pulmonary alveolar type II cells. Biochem. Pharmacol..

[47] Galm U., Hager M.H., Van Lanen S.G., Ju J., Thorson J.S., Shen B. (2005). Antitumor antibiotics: Bleomycin, enediynes, and mitomycin. Chem. Rev..

[48] Cheresh P., Kim S.J., Tulasiram S., Kamp D.W. (2013). Oxidative stress and pulmonary fibrosis. Biochim. Biophys. Acta.

[49] Galuppo M., Di Paola R., Mazzon E., Esposito E., Paterniti I., Kapoor A., Thiemermann C., Cuzzocrea S. (2010). GW0742, a high affinity PPAR-beta/delta agonist reduces lung inflammation induced by bleomycin instillation in mice. Int. J. Immunopathol. Pharmacol..

[50] Di Paola R., Talero E., Galuppo M., Mazzon E., Bramanti P., Motilva V., Cuzzocrea S. (2011). Adrenomedullin in inflammatory process associated with experimental pulmonary fibrosis. Respir. Res..

[51] Galuppo M., Esposito E., Mazzon E., Di Paola R., Paterniti I., Impellizzeri D., Cuzzocrea S. (2011). MEK inhibition suppresses the development of lung fibrosis in the bleomycin model. Naunyn Schmiedebergs Arch. Pharmacol.

[52] Lakari E., Soini Y., Saily M., Koistinen P., Paakko P., Kinnula V.L. (2002). Inducible nitric oxide synthase, but not xanthine oxidase, is highly expressed in interstitial pneumonias and granulomatous diseases of human lung. Am. J. Clin. Pathol..

[53] Valentino L., Pierre J. (2006). JAK/STAT signal transduction: Regulators and implication in hematological malignancies. Biochem. Pharmacol..

[54] Zhang Y., Dees C., Beyer C., Lin N.Y., Distler A., Zerr P., Palumbo K., Susok L., Kreuter A., Distler O. (2015). Inhibition of casein kinase II reduces TGFbeta induced fibroblast activation and ameliorates experimental fibrosis. Ann. Rheum. Dis..

[55] Tian B., Patrikeev I., Ochoa L., Vargas G., Belanger K.K., Litvinov J., Boldogh I., Ameredes B.T., Motamedi M., Brasier A.R. (2017). NF-κB mediates mesenchymal transition, remodeling, and pulmonary fibrosis in response to chronic inflammation by viral RNA patterns. Am. J. Resp. Cell Mol..

[56] Bowie A., O’Neill L.A. (2000). Oxidative stress and nuclear factor-kappaB activation: A reassessment of the evidence in the light of recent discoveries. Biochem. Pharmacol..

[57] Selman M., King T.E., Pardo A., American Thoracic S., European Respiratory S., American College of Chest P. (2001). Idiopathic pulmonary fibrosis: Prevailing and evolving hypotheses about its pathogenesis and implications for therapy. Ann. Intern. Med..

[58] Moon T.C., St Laurent C.D., Morris K.E., Marcet C., Yoshimura T., Sekar Y., Befus A.D. (2010). Advances in mast cell biology: New understanding of heterogeneity and function. Mucosal. Immunol..

[59] Inoue Y., King T.E., Tinkle S.S., Dockstader K., Newman L.S. (1996). Human mast cell basic fibroblast growth factor in pulmonary fibrotic disorders. Am. J. Pathol..

[60] Kawanami O., Ferrans V.J., Fulmer J.D., Crystal R.G. (1979). Ultrastructure of pulmonary mast cells in patients with fibrotic lung disorders. Lab. Investig..

[61] Cha S.I., Chang C.S., Kim E.K., Lee J.W., Matthay M.A., Golden J.A., Elicker B.M., Jones K., Collard H.R., Wolters P.J. (2012). Lung mast cell density defines a subpopulation of patients with idiopathic pulmonary fibrosis. Histopathology.

[62] Andersson C.K., Andersson-Sjoland A., Mori M., Hallgren O., Pardo A., Eriksson L., Bjermer L., Lofdahl C.G., Selman M., Westergren-Thorsson G. (2011). Activated MCTC mast cells infiltrate diseased lung areas in cystic fibrosis and idiopathic pulmonary fibrosis. Respir. Res..

[63] Edwards S.T., Cruz A.C., Donnelly S., Dazin P.F., Schulman E.S., Jones K.D., Wolters P.J., Hoopes C., Dolganov G.M., Fang K.C. (2005). c-Kit immunophenotyping and metalloproteinase expression profiles of mast cells in interstitial lung diseases. J. Pathol..

[64] Overed-Sayer C., Rapley L., Mustelin T., Clarke D.L. (2013). Are mast cells instrumental for fibrotic diseases?. Front. Pharmacol..

[65] Zhao X.Y., Zhao L.Y., Zheng Q.S., Su J.L., Guan H., Shang F.J., Niu X.L., He Y.P., Lu X.L. (2008). Chymase induces profibrotic response via transforming growth factor-beta 1/Smad activation in rat cardiac fibroblasts. Mol. Cell Biochem..

[66] Lindstedt K.A., Wang Y., Shiota N., Saarinen J., Hyytiainen M., Kokkonen J.O., Keski-Oja J., Kovanen P.T. (2001). Activation of paracrine TGF-beta1 signaling upon stimulation and degranulation of rat serosal mast cells: A novel function for chymase. FASEB J..

[67] Fernandez I.E., Eickelberg O. (2012). The impact of TGF-β on lung fibrosis: From targeting to biomarkers. Proc. Am. Thorac. Soc..

[68] Sime P.J., Xing Z., Graham F.L., Csaky K.G., Gauldie J. (1997). Adenovector-mediated gene transfer of active transforming growth factor-beta1 induces prolonged severe fibrosis in rat lung. J. Clin. Investig..

[69] Tanaka K.-I., Ishihara T., Azuma A., Kudoh S., Ebina M., Nukiwa T., Sugiyama Y., Tasaka Y., Namba T., Ishihara T. (2010). Therapeutic effect of lecithinized superoxide dismutase on bleomycin-induced pulmonary fibrosis. Am. J. Physiol. Lung Cell. Mol. Physiol..

[70] Sims J.E., Smith D.E. (2010). The IL-1 family: Regulators of immunity. Nat. Rev. Immunol..

[71] Tatler A.L., Jenkins G. (2012). TGF-beta activation and lung fibrosis. Proc. Am. Thorac. Soc..

